# Switching from allopurinol to febuxostat: efficacy and safety in the treatment of hyperuricemia in renal transplant recipients

**DOI:** 10.1080/0886022X.2019.1632717

**Published:** 2019-07-03

**Authors:** Yanchun Li, Min Liu, Xuelei Zhang, Yuewu Lu, Juan Meng

**Affiliations:** a Department of Nephrology, Beijing Chao-Yang Hospital, Capital Medical University, Beijing, PR China;; b Department of Rheumatology and Immunology, Beijing Chao-Yang Hospital, Capital Medical University, Beijing, PR China

**Keywords:** Hyperuricemia, renal transplant, febuxostat, allopurinol

## Abstract

The aim of this study was to evaluate the efficacy and tolerability of febuxostat in renal transplant recipients who were previously treated with allopurinol (the daily oral dose is 100 mg). A 6-month cohort study was conducted with 46 renal transplant recipients who had hyperuricemia. In 22 patients, treatment was changed from allopurinol to febuxostat (febuxostat was given at an oral dose of 20 mg once a day), and the other 24 patients continued the allopurinol treatment (the daily oral dose is 100 mg). The serum levels of uric acid (UA), creatinine, other biochemical parameters, estimated glomerular filtration rate (eGFR), and adverse events were measured at baseline as well as at 1, 3, and 6 months after the switch to febuxostat. Serum UA levels significantly decreased from 470.82 ± 34.37 to 378.77 ± 51.97 μmol/L in the febuxostat group, and decreased from 469.46 ± 33.47 to 428.21 ± 23.37 μmol/L in the allopurinol group. The eGFR increased from 75.55 to 85.23 mL/min in the febuxostat group, and decreased from 78.79 to 70.31 mL/min in the allopurinol group. In renal transplant recipients, febuxostat reduced the serum UA levels resulting in minor short-term improvement of renal function with no changes in the other biochemical parameters.

## Introduction

Hyperuricemia is a frequent complication after renal transplantation, marked by having a serum uric acid (UA) concentration greater or equal to 7.0 mg/dL (417 μmol/L) in men and 6.0 mg/dL (357 μmol/L) in women [1,2]. The prevalence of hyperuricemia ranges from 19 to 55% in patients whose immunosuppressive regimen did not include cyclosporin A (CsA) and from 30 to 84% in patients treated with CsA in renal transplant recipients [3]. Many factors contribute to hyperuricemia, including insufficient allograft function, treatment with diuretics, immunosuppression with calcineurin inhibitor (CNI), and immunosuppression with mizoribine [4–6].

In the general population, asymptomatic hyperuricemia is a risk factor for the onset and progression of chronic kidney disease and for cardiovascular disease [7,8]. Additionally, hyperuricemia is associated with allograft loss and onset of cardiovascular disease in renal transplant recipients [8–10].

Allopurinol is a first-line medication commonly used to treat gout and/or hyperuricemia [11]. However, allopurinol is associated with multiple side-effects, including increased toxicity with low glomerular filtration rate resulting in increased risk of allopurinol hypersensitivity syndrome (AHS), hepatotoxicity, and Stevens-Johnson syndrome. Febuxostat is a new nonpurine selective xanthine oxidase inhibitor well tolerated in patients with Gout and in those with mild or moderate chronic kidney disease [12–15]. However, the long-term efficacy and tolerability of febuxostat after switching from allopurinol in renal transplant recipients have not been assessed. The aim of this study was to evaluate the efficacy and safety of switching from allopurinol to febuxostat in management of hyperuricemia and the progression of renal function in stable renal transplant recipients.

## Methods

### Study design

We performed a 6-month observational study of 100 renal transplant recipients who visited Beijing Chao-Yang Hospital between July 2016 and July 2017. Hyperuricemia was treated with allopurinol for 3 months and the time after transplantation exceeded 6 months. Immunosuppressive agents were not switched during the study.

A total of 46 renal transplant recipients in our clinic had hyperuricemia and were treated with allopurinol at a dose of 100 mg/day at first visit. Febuxostat was prescribed for 22 patients of 46 patients, who switched from allopurinol at 100 mg/day to febuxostat at 20 mg/day. These 22 patients constituted the febuxostat group. The other 24 patients continued treatment with allopurinol at 100 mg/day and constituted the allopurinol group.

### Enrollment and exclusion criteria

Enrolled subjects were all recipients with stable renal function and serum creatinine levels below 176.8 μmol/L (2 mg/dL). The criteria for exclusion for the study included: (a) the patients who received allopurinol at a dose of 50 or 150, or 200 mg/day, (b) a history of active liver disease, and (c) stable clinical condition (no hospitalization in the previous 3 months). The ethics committee of Beijing Chao-Yang Hospital approved the study’s protocols, patient information, and informed consent forms (12012).

### Determination

The baseline demographic data, laboratory data, and information concerning comorbid conditions and medication were collected during the first clinical visit. We defined hyperuricemia as uric acid (UA) ≥420 μmol/L in males and UA ≥ 360 μmol/L in females with or without treatment. Serum levels of UA, creatinine and other biochemical parameters were measured at baseline as well as at 1, 3 and 6 months after switching to febuxostat therapy. The estimated glomerular filtration rate (eGFR) was calculated using a modified estimate for Chinese population: [eGFR (mL/min/1.73 m^2^) =175 × SCr (mg/dL)^−1.234^ × age^−0.179^ (×0.79 if women)] [16].

### Statistical analysis

Continuous variables are reported as the mean ± SD, and categorical variables are reported as percentages unless otherwise stated. Non-paired two-tailed student’s *t* tests were used to compare continuous variables between the febuxostat group and allopurinol group. Paired two-tailed student’s *t* tests were used to compare pretreatment and post-treatment values. *p* Values less than 0.05 were considered to indicate statistical significance. Results were analyzed using the SPSS 19.0 software (SPSS Inc.).

## Results

### Baseline characteristics of patients in the febuxostat group and allopurinol group

Forty-six renal transplant recipients were enrolled in the study, of which 41 (89%) were male and 5 (11%) were female. The average age was 43.96 ± 13.02 years (range, 21–71 years). 22 patients switched from allopurinol to febuxostat, and the other 24 patients continued their allopurinol treatment during the 6-month observation period. The baseline characteristics, including laboratory data from both groups are shown in Table 1. All other medications were continued with no dosage during the 6-month observational period. There were no significant differences in clinical parameters in the febuxostat group and allopurinol group.

**Table 1.** Baseline characteristics of patients in the febuxostat group and allopurinol group.

	Febuxostat group (*n* = 22)		Allopurinol group (*n* = 24)	*p* Value
Age (years)	43.82 ± 12.63		44.08 ± 13.65	0.947
Gender (M:F)	20:2		21:3	0.927
BMI (kg/m^2^)	21.87 ± 3.17		21.75 ± 2.39	0.885
MAP (mmHg)	88.64 ± 8.89		89.79 ± 9.52	0.675
Hemoglobin (g/dL)	12.02 ± 1.3		12.18 ± 1.36	0.686
Serum albumin (g/L)	38.82 ± 3.97		38.96 ± 3.83	0.904
Serum creatinine (mg/dL)	1.13 ± 0.3		1.11 ± 0.31	0.826
eGFR (mL/min per 1.73 m^2^)	75.55 ± 25.73		78.79 ± 28.55	0.689
Time after transplantation (years)	4.34 ± 1.18		4.34 ± 1.69	0.633
Uric acid (umol/L)	470.82 ± 34.37		469.46 ± 33.47	0.893
Etiology of renal disease (%)				
Glomerulonephritis	8		9	0.961
Nephrosclerosis	4		3	
Polycystic kidney disease	3		5	
Diabetes mellitus	3		3	
Others	4		4	
Immunosuppressant therapy, *n* (%)				
Tacrolimus	9		7	0.871
Mycophenolate mofetil	11		10	
Prednisolon	10		11	
Other therapy, *n* (%)				
ARB treatment	5		4	0.927
CCB treatment	6		4	
PPI treatment	12		11	
Statin treatment	8		9	

ARB: angiotensin receptor blocker; BMI: body mass index; CCB: calcium channel blocker; eGFR: estimated glomerular filtration rate; MAP: mean blood pressure; PPI: proton pump inhibitor.

### Effect of the switch from allopurinol to febuxostat on serum UA levels

Table 2 shows the effect of switching from allopurinol to febuxostat on serum UA levels. The ratio of patients with hyperuricemia at baseline in the febuxostat group and allopurinol group was 90.9 and 91.7%, respectively (*p* = 0.927). The ratio of patients with hyperuricemia at 6 months in the febuxostat group and allopurinol group was 18.2 and 70.8%, respectively (*p* < 0.001). The serum UA level was significantly higher at baseline (464.32 ± 39.82 μmol/L) than at 1, 3, and 6 months, respectively (420.95 ± 45.75, 395.41 ± 46.92, and 378.77 ± 51.97 μmol/L, respectively; all *p* < 0.05). Febuxostat dosage was reduced to 10 mg/day at 6 months in one patient because serum UA level had decreased to 240 from 370 μmol/L. Febuxostat dosage was not changed in other patients during the observation period.

**Table 2.** Effect of the switch from allopurinol to febuxostat on serum UA levels.

	Febuxostat group (*n* = 22)	Allopurinol group (*n* = 24)	*p* Value
Before treatment	470.82 ± 34.37	469.46 ± 33.47	0.893
Basal	464.32 ± 39.82	462.46 ± 40.33	0.876
1 months	420.95 ± 45.75	448.71 ± 34.43	0.024*
3 months	395.41 ± 46.92	437.63 ± 32.03	0.001**
6 months	378.77 ± 51.97	428.21 ± 23.37	0.001**

* *p* < 0.05 compared with allopurinol group. **p < 0.01 compared with allopurinol group.

### Effect of the switch from allopurinol to febuxostat on estimated glomerular filtration rate (eGFR)

Table 3 shows the effect of switching from allopurinol to febuxostat on eGFR. The eGFR level of febuostat group was significantly higher at 6 months than allopurinol group (85.23 ± 20.24 and 70.31 ± 24.51 mL/min, *p* = 0.03). The eGFR level was higher at 1, 3and 6 months, respectively (81.52 ± 22.27, 84.03 ± 20.84, and 85.23 ± 20.24 mL/min, respectively; but all *p* > 0.05) than at baseline (75.55 ± 25.73 mL/min).

**Table 3.** Effect of the switch from allopurinol to febuxostat on eGFR.

	Febuxostat group (*n* = 22)	Allopurinol group (*n* = 24)	*p* Value
Before treatment	76.73 ± 22.42	74.67 ± 21.35	0.751
Basal	75.55 ± 25.73	78.79 ± 28.55	0.689
1 months	81.52 ± 22.27	78.38 ± 27.6	0.675
3 months	84.03 ± 20.84	76.22 ± 23.58	0.242
6 months	85.23 ± 20.24	70.31 ± 24.51	0.03*

* *p* < 0.05 compared with allopurinol group.

### Efficacy of the switch from allopurinol to febuxostat during the observation period

Table 4 shows the efficacy of switching from allopurinol to febuxostat. No serious adverse events were recorded and liver function tests were not altered by febuxostat. None of the 22 patients in the febuxostat group experienced severe adverse effects, such as gout attacks or skin rash. Serum alanine amino-transferase, aspartate aminotransferase, alkaline phosphatase, and total bilirubin concentrations were similar at baseline, 1, 3, and 6 months.

**Table 4.** Safety of the switch from allopurinol to febuxostat during the observation period.

	Baseline	Observation period			
		1	3	6	*p* Value
Hemoglobin (g/dL)	120.18 ± 13.02	121.95 ± 10.49	122.82 ± 9.07	122.91 ± 7.9	0.802
Serum albumin (g/L)	38.82 ± 3.97	39.91 ± 2.88	39.86 ± 2.96	39.95 ± 2.79	0.589
Aspartate transaminase (U/L)	23.64 ± 7.29	22.86 ± 6.8	24.22 ± 6.78	24.77 ± 6.35	0.813
Alanine aminotransferase (U/L)	23 ± 6.98	23.95 ± 7.08	24.91 ± 6.76	25.77 ± 5.68	0.547
Alkaline phosphatase (U/L)	97.95 ± 24.83	87.95 ± 24.83	86.68 ± 25.4	88.09 ± 24.58	0.412
Lactate dehydrogenase (U/L)	186.32 ± 31.33	186.09 ± 31.55	187.91 ± 31.24	186.77 ± 30.94	0.998
γ-glutamyl transpeptadase (U/L)	22.27 ± 8.08	22.41 ± 6.18	23.64 ± 5.49	24.36 ± 4.41	0.63
Total bilirubin (Umol/L)	16.68 ± 2.55	17.41 ± 2.95	17.23 ± 2.81	17.18 ± 2.74	0.839

* *p* < 0.05 between baseline and 1, 3, and 6 months.

## Discussion

Hyperuricemia is a frequent complication of renal transplant recipients. A large epidemiologic study of 177,570 individuals for a total of 527,597 person-years revealed that a higher serum UA levels was an independent risk factor for end-stage renal disease (HR 2.14 [1.65–2.77] for highest versus lowest quartile) [17]. Results from the meta-analysis also suggest that UA-lowering therapy may have efficacy for delaying progression of CKD [18]. Elevated UA was significantly associated with rapid kidney function decline and incident CKD particularly in the community-based cohort of African Americans from the Jackson Heart Study [19,20]. According to the previous study by Haririan A et al., the mean UA levels in renal recipients is an independent predictor of long-term graft survival and short-term graft function [21]. It also has been reported that hyperuricemia is an independent risk factor for pediatric renal transplant recipients [22].

Febuxostat was shown to lower serum UA levels significantly in renal transplant recipients. Sofue et al. [23] showed the efficacy and safety of using (10–20 mg/day) febuxostat in renal transplant recipients. Bayram et al. [24] hypothesized that lowering UA levels with allopurinol therapy seemed to help restore endothelial function and slow the progression of CKD. After 1 year of treatment, the majority of treated patients had achieved target serum UA levels and stabilized eGFR. Our study resulted with reduced serum UA levels in renal transplant recipients without reducing the doses and type of immunosuppressants after switching from allopurinol to febuxostat. Serum UA levels were 395.41 ± 46.92 and 378.77 ± 51.97 μmol/L at 3 and 6 months after the switch, respectively.

Previous studies postulate the causal role of UA in the progression of renal disease and cardiovascular diseases. Mallat et al. and Kuwabara et al. have found that hyperuricemia could induce pathological restructuring of vessels and vascular nephrosclerosis, and was associated with the mortality and development of hypertension, cardiovascular diseases, and chronic renal diseases, [25,26] while risk factors for CKD and hyperuricemia include gender, alcohol consumption, BMI and other characteristics [27]. It has been reported recently that soluble UA has important biological roles such as in pro-inflammatory and proliferative effects on vascular smooth muscle cells; induction of the dysfunction of endothelial cells in rats; and in inducing systemic inflammatorome and generation of oxidative stress [28,29].

Allopurinol has long been regarded as the only XO inhibitor drug. Since allopurinol is metabolized in the kidney, the dosage must be reduced in patients with renal diseases and in renal transplant recipients. By contrast, since febuxostat is metabolized by the kidney and liver, it is not necessary to reduce the dosage in patents with renal disease. Some studies have demonstrated that the risk of allopurinol-induced adverse reactions has increased in renal diseases patients [30]. As shown in Table 4, clinical laboratory data showed no significant changes during observation period from baseline to 6 months after the switch to febuxostat. No serious adverse events were recorded and liver function tests were not altered by febuxostat. Therefore, this study suggests that febuxastat is a more efficacious therapy than allopurinol.

This study was associated with several limitations. First, the data was obtained retrospectively from a relative small patient population in a 6-month study located in a single clinic. The evaluation of the small sample size and unmatched groups can introduce bias. Well-designed prospective studies are required to confirm our findings. Second, the follow-up duration was short, and we did not question patients on their diet or other characteristics that may confound our results. Also, urate-lowering therapy in renal transplant recipients has many potential confounding factors.
